# Mechanical and Structural Performance of Bio-Resin Composites Reinforced with Biopolymer Nonwoven Fabrics

**DOI:** 10.3390/polym18101215

**Published:** 2026-05-16

**Authors:** Anna Sowińska-Baranowska, Marcin Masłowski, Justyna Miedzianowska-Masłowska, Magdalena Maciejewska, Dainius Martuzevičius, Tadas Prasauskas, Goda Masione

**Affiliations:** 1 Department of Chemistry, Institute of Polymer and Dye Technology, Lodz University of Technology, Stefanowskiego Street 16, 90-537 Lodz, Poland; justyna.miedzianowska@p.lodz.pl (J.M.-M.); magdalena.maciejewska@p.lodz.pl (M.M.); 2 Faculty of Chemical Technology, Kaunas University of Technology, Radvilenų pl. 19 C, 50254 Kaunas, Lithuania; dainius.martuzevicius@ktu.lt (D.M.); tadas.prasauskas@ktu.lt (T.P.); goda.masione@ktu.lt (G.M.)

**Keywords:** biocomposites, bio-based resins, nonwoven fabrics, electrospinning, melt-blown, mechanical properties, interfacial adhesion, PLA, PBS, PA1010

## Abstract

This study investigates the mechanical, structural, and thermal performance of bio-based composite laminates reinforced with nonwoven fibrous materials derived from polylactic acid (PLA), poly(butylene succinate) (PBS), and polyamide 1010 (PA1010). The fibrous reinforcements, produced using melt-blown and electrospinning techniques, were characterized in terms of morphology, fibre diameter distribution, and wettability, and subsequently incorporated into bio-resin laminates to strengthen them. The curing behaviour of the composites was evaluated using differential scanning calorimetry (DSC). The results demonstrate that fibre structure and morphology strongly influence resin impregnation and interfacial interactions. Mechanical properties varied significantly depending on the reinforcement type. PA1010-based laminates exhibited the highest strength and stiffness due to their compact and uniform fibrous structure. PBS-based systems showed intermediate behaviour, while PLA-based composites displayed lower strength but higher deformability. DSC results indicated that fibre type affected crosslinking efficiency. Thermogravimetric analysis (TGA) revealed similar initial thermal stability of laminates (T_5_% ≈ 299–313 °C), governed by the resin matrix, while differences at higher temperatures reflected the type of reinforcement used. These findings highlight the importance of fibre morphology and interfacial compatibility in designing sustainable composite laminates reinforced with recycled fibrous materials.

## 1. Introduction

In recent years, increasing attention has been devoted to the development of polymeric materials derived from renewable resources. Bio-based polymers and bio-resins are considered promising alternatives to conventional petroleum-derived materials due to their reduced environmental impact and compatibility with sustainable material design strategies [1,2]. Additionally, in the face of growing environmental protection challenges and the need to implement circular economy principles, the search for innovative methods of managing industrial waste is becoming increasingly important. One such waste stream is spent filter fabrics, which, after completing their service life, are most often disposed of in landfills or incinerated, despite still possessing valuable mechanical and structural properties [3,4,5]. Their fibrous structure, strength, and resistance to chemical agents make them a potential secondary raw material for advanced material applications. Simultaneously, a dynamic development of composite materials based on bio-resins can be observed, as they constitute more environmentally friendly alternatives to conventional synthetic resins. Bio-resins, derived from renewable resources, contribute to reducing the carbon footprint; however, they often require appropriate reinforcement to achieve the desired mechanical properties [6,7]. In this context, the use of spent filter fabrics as a reinforcing component in composite laminates appears to be a solution that is both materially efficient and environmentally beneficial. The use of this type of waste as reinforcement in composite laminates aligns with the concept of upcycling, which involves converting waste materials into products of higher added value. The incorporation of spent filter fabrics into the composite structure may contribute to the improvement of its mechanical properties, such as tensile strength, flexural strength, and fracture resistance, while simultaneously reducing the consumption of virgin reinforcing materials. However, it is crucial to assess the material compatibility between the bio-resin and the fibrous reinforcement, as well as its influence on the properties of the final products. In the context of designing composites based on bio-resins and secondary fibrous reinforcements, such as spent filter fabrics, the selection of an appropriate polymer matrix is of key importance. Among materials aligned with the principles of sustainable development, particular attention is given to biodegradable or partially bio-based polymers. Biopolymers represent a heterogeneous group of materials that can be classified according to their origin and synthesis pathway [8,9]. From a production perspective, they include: naturally occurring polymers derived from biomass, polymers produced by microorganisms (e.g., PHAs), and bio-based polymers synthesized chemically from renewable feedstocks, yet structurally equivalent to their petroleum-based counterparts, such as PE, PP, PBS, PA, and PLA [8,10,11].

Polylactic acid (PLA) is one of the most widely utilized biopolymers, derived from renewable resources such as corn starch and sugarcane [12]. It is characterized by relatively high stiffness and tensile strength [13,14], making it an attractive candidate for use as a matrix in composite materials [15]. However, PLA also exhibits inherent brittleness and limited impact resistance [16,17], which may significantly restrict its applicability in structural applications. Furthermore, its glass transition temperature, typically in the range of 55–65 °C, indicates limited thermal stability at elevated temperatures [18]. From the perspective of compatibility with fibre reinforcement, PLA generally exhibits good wettability toward fibre surfaces; however, due to limited interfacial adhesion and intrinsic brittleness, it often requires modification (e.g., plasticization, fibre surface treatment, or compatibilizing additives) to enhance interfacial bonding and improve the fracture resistance of the composite [19,20].

Poly(butylene succinate) (PBS) is a biodegradable aliphatic polyester characterized by higher flexibility and impact resistance than PLA, with mechanical properties comparable to conventional polyolefins such as polyethylene and polypropylene [21,22,23,24]. Its low glass transition temperature (~30 °C) and melting temperature (110–120 °C) ensure good processability, while its ductility makes it effective in reducing the brittleness of fibre-reinforced composites [22,23,24].

Polyamide 1010 (PA1010) is a bio-based engineering polymer, produced, among others, from castor oil. Unlike PLA and PBS, it is not biodegradable; however, its renewable origin aligns with strategies aimed at reducing reliance on petrochemical resources [25]. PA1010 is characterized by high mechanical strength, good abrasion resistance, and favourable tribological properties [25,26]. It also exhibits high chemical resistance and relatively low moisture absorption compared to other polyamides [26,27]. Its melting temperature is approximately 200–210 °C, which enables applications in more demanding thermal conditions. Owing to its ductility and resistance to cracking, PA1010 can serve as an effective matrix for fabric-reinforced composites, ensuring efficient stress distribution and enhanced durability of the final material [28].

A comparison of the properties of PLA, PBS, and PA1010 indicates that each of these materials can play a distinct role in the design of biocomposites. PLA provides high stiffness, PBS offers flexibility and impact resistance, whereas PA1010 delivers advanced mechanical and service performance. The final selection of the matrix should take into account both the application requirements and the characteristics of the applied secondary reinforcement, including its structure, degree of wear, and compatibility with the polymer. Previous studies by Masione et al. [29] described in detail the methods for manufacturing the fabrics from bio-based polymers, including PLA, PBS and PA1010, along with their structural and mechanical characteristics. In the present work, as a continuation of that research, these fabrics were reused and incorporated as reinforcing fibres in laminates based on bio-derived resins. Bio-based laminates, i.e., composites produced using resins derived from renewable resources instead of petrochemical sources, are gaining increasing attention due to their ability to combine favourable mechanical performance with a reduced environmental impact. As a result, they are being widely explored and applied across various industrial sectors, including advanced technologies, automotive engineering, and transportation. Bio-based resins usually reach about 90–95% of the strength of traditional epoxy resins, while still meeting the requirements of most applications and providing a strong compromise between performance and environmental sustainability [30]. Despite their advantages, such as sustainable sourcing of bio-resins, abundant availability, and structural tunability, bio-based resins derived from natural resources exhibit certain inherent limitations, which may restrict their performance compared to conventional petroleum-based epoxy resins [30]. To address these performance limitations, current research has mainly concentrated on several modification strategies. These include increasing the crosslinking density by introducing rigid molecular structures, i.e., lignin and cellulose derivatives or highly functional curing agents (e.g., rosin-based anhydrides). Moreover, improving mechanical performance and thermal stability is achieved through the incorporation of inorganic fillers, i.e., nanosilica, as well as carbon-based reinforcements, i.e., carbon nanotubes and functionalized graphene. An additional approach is enhancing flame resistance of such composites by applying flame-retardant systems based on nitrogen, phosphorus, silicon, halogens, or by using transition metal oxides [30]. However, these modification strategies are associated with various challenges and techno-economic limitations that significantly restrict the broader application of bio-resin-based materials [30]. Accordingly, there is a strong need to pursue new modification approaches that are economically viable, environmentally responsible, and scalable, as this is crucial for enabling the wider practical implementation of bio-based thermosetting resins. Therefore, the aim of this study is not only to evaluate the performance of bio-resin composites reinforced with recycled biopolymer nonwoven fabrics, but also to establish a structure–property relationship linking fibre morphology (diameter distribution and structural organization), wettability, curing behaviour, and mechanical performance. By correlating these parameters, this work provides a design-oriented framework for the development of sustainable composite laminates based on secondary fibrous reinforcements.

## 2. Materials and Methods

### 2.1. Materials

**Figure 1:**
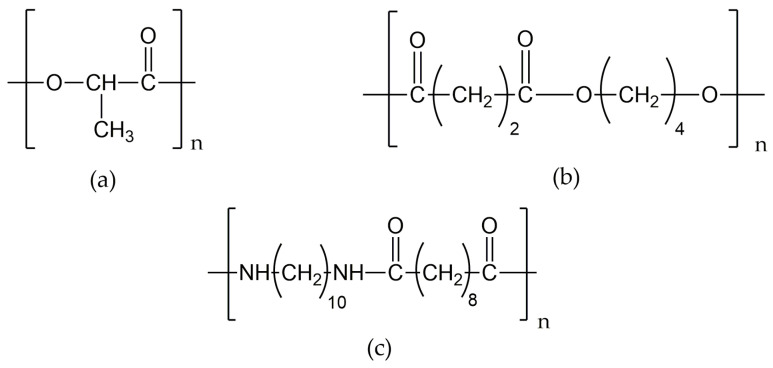
The structure of bio-based polymers: (**a**) polylactic acid, PLA; (**b**) polybutylene succinate, PBS; (**c**) polyamide 1010, PA1010.

Nano- and microfibrous air filtering materials were fabricated from synthetic biobased polymers, including polybutylene succinate (PBS), polyamide 1010 (PA1010), and polylactic acid (PLA). The materials were produced as nonwoven fabrics with controlled nano- and microscale fibre diameters, providing high specific surface area and tailored porosity. The fibrous mats contained different weight fractions of the aforementioned polymers, as described by Masione et al. [8,29]. Their structural formulas are shown in Figure 1.

The fibrous structures were designed according to the procedure reported by the authors, using a combination of electrospinning and melt-blown techniques. This approach enabled the formation of multilayer structures integrating nanofibrous layers—responsible for high filtration efficiency with microfibrous layers—providing mechanical strength and low airflow resistance. The resulting fibrous materials were used as a reinforcement in composite laminates.

A bio-based epoxy laminating resin (Entropy Resins, ONE system, California, CA, USA) was used as the composite matrix, consisting of a base component (Part A) and a hardener (Part B). The resin is characterized by a high content of renewable raw materials and reduced emissions of volatile organic compounds (VOCs), making it suitable for environmentally friendly applications. The reference sample corresponds to the neat bio-based epoxy resin (without fibrous reinforcement), cured under identical conditions as the composites.

### 2.2. Methods

#### 2.2.1. Preparation of Fibrous Reinforcements and Composite Laminates

Nano- and microfibrous filtering materials were fabricated using electrospinning and conventional techniques (e.g., melt-blown), depending on the type of polymer. The polymers were applied in varying weight fractions. Detailed preparation procedures of the fibrous materials are described elsewhere [8,29].

Composite laminates were fabricated using a hand lay-up method. Nonwoven fibrous materials (PLA, PBS and PA1010) were used as a reinforcement and arranged in layers within the mould. The bio-based epoxy resin was mixed with the hardener according to the manufacturer’s recommended stoichiometric weight ratio (2:1 by weight) to minimize air entrapment. The resulting mixture was then applied to the fibrous layers to ensure complete impregnation. Curing was carried out at room temperature for 24 h. The composite laminates were obtained as multilayer structures consisting of stacked nonwoven fibrous mats impregnated with the bio-resin system. The final laminate thickness ranged from approximately 1 to 2 mm (±0.2 mm), depending on the type of reinforcement and fibre packing density. All laminates were fabricated using the same procedure to ensure consistency and reproducibility between samples.

#### 2.2.2. Characterization of Fibrous Fabrics and Bio-Resin Composites

Thermogravimetric analysis (TGA) was conducted to evaluate the thermal stability of fabrics. Measurements were performed using a TGA/DSC1 system (Mettler Toledo, Greifensee, Switzerland). Samples were heated from 25 °C to 900 °C at a rate of 10 °C/min under argon (50 mL/min) up to 600 °C, followed by air (50 mL/min) up to 900 °C. The same method was used for bio-based composites studies.

Optical microscopy observations of the fabric’s structure were carried out using an Optatech optical microscope (Optatech, Warsaw, Poland) at a magnification of ×200. Images were captured with a Leica MZ 6 camera (Leica Microsystems, Wetzlar, Germany) and processed using OptaView 7 software (Optatech, Warsaw, Poland).

The wettability of fabrics was evaluated by contact angle (CA) measurements using a goniometer (DataPhysics Instruments, OCA 15EC, Filderstadt, Germany). A droplet of liquid (1–2 µL) was deposited onto the surface of each fabric and also bio-resin, to determine the contact angle. Measurements were conducted using distilled water as a probe liquid. Each sample was analyzed in six repetitions to ensure the reliability and reproducibility of the results.

Differential scanning calorimetry (DSC) analysis was performed in accordance with ISO 11357-1 using a DSC1 instrument from Mettler Toledo (Greifensee, Switzerland) to evaluate the curing behaviour of the bio-resin and to determine the enthalpy of the curing reactions. Bio-resin samples of approximately 75 mg were sealed in 100 µL aluminum crucibles and heated from 25 °C to 200 °C at a rate of 10 °C/min under an argon atmosphere. The DSC data were analyzed using STARe software (version 16.40).

The tensile behaviour of the bio-resin composites was evaluated under quasi-static loading using a universal testing machine (Zwick/Roell 1435, Ulm, Germany). The testing procedure was based on the general principles of ISO 527-1 [31]; however, due to the laminate manufacturing process, non-standard rectangular specimens were used instead of type 1A dumbbell specimens. The test specimens were cut from composite laminates in the form of rectangular strips with a length of approximately 60 mm, a width of 10 mm, and a thickness in the range of 1–2 mm (±0.2 mm). All samples were carefully prepared to ensure high-dimensional repeatability. Prior to testing, the actual width and thickness within the gauge length were measured at three randomly selected locations using a digital calliper with a resolution of 0.01 mm, and mean values were used for stress calculations. At least four independent specimens were tested for each material variant.

It should be noted that, due to the laminate fabrication method and limited panel dimensions, standard specimen geometries (e.g., type 1A) could not be obtained. Therefore, the results should be interpreted primarily in a comparative manner between the investigated systems rather than as absolute values. 

The hardness of the bio-resin composites was determined using Shore D indentation in accordance with ISO 868 [32]. Measurements were carried out using a Zwick 3105 durometer (Ulm, Germany), equipped with a conical indenter (30° tip angle, 0.1 mm tip radius), suitable for hard polymeric materials and composite laminates.

The flexural properties of the bio-resin composites were determined using a three-point bending test performed on a Zwick/Roell RetroLine universal testing machine (Ulm, Germany) equipped with a bending fixture. The testing methodology followed the general guidelines of ISO 178 [33]; however, specimens of non-standard geometry were used. The test specimens were prepared by cutting rectangular strips from the fabricated laminates, with dimensions of approximately 60 mm in length, 10 mm in width, and 1–2 mm in thickness. Prior to testing, specimen dimensions were measured using a digital calliper and used in subsequent calculations. A minimum of three specimens were tested for each material variant. Similarly, due to the limitations related to laminate dimensions, non-standard specimen geometry was applied. All specimens were prepared with comparable dimensions and tested under identical conditions; therefore, the results are intended for comparative analysis between the studied materials.

## 3. Results

### 3.1. Thermogravimetric Analysis of Fabrics

**Figure 2:**
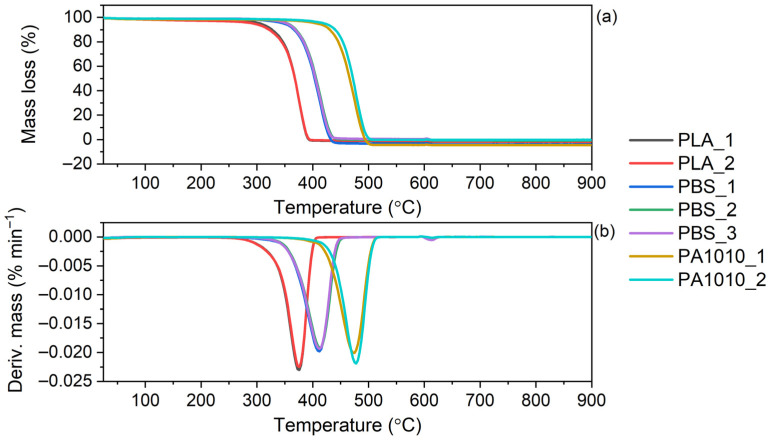
Thermal stability of neat fabrics: (**a**) TG curves, (**b**) DTG curves.

Thermogravimetric analysis (TGA) of the neat fabrics was carried out, and the corresponding TG and DTG curves are presented in Figure 2. The results are summarized in Table 1.

The thermogravimetric analysis (TGA) results of the fabrics based on PLA, PBS and PA1010 revealed significant differences in their thermal stability, as shown in Table 1 and illustrated in Figure 2. The TG and derivative thermogravimetric (DTG) curves demonstrated that all investigated materials underwent complete and predominantly single-step degradation, as evidenced by a single pronounced peak on DTG curves, the absence of residual mass at higher temperatures and no residue at 900 °C left after the decomposition process. The onset of decomposition, expressed as T_5%_, clearly indicated that PLA exhibited the lowest thermal resistance (298–305 °C), followed by PBS (348–355 °C), while PA1010 showed the highest thermal stability (406–420 °C). A similar trend was observed for T_10%_ and T_50%_, confirming that the thermal stability increased in the order of PLA < PBS < PA1010, which is consistent with expectations based on the inherent thermal stability of the respective polymers. The thermal degradation behaviour of neat PLA, PBS, and PA1010 has been widely reported in the literature and provides a useful reference for interpreting the results obtained in this study. Neat PLA is characterized by relatively low thermal stability, with the main degradation occurring in the range of approximately 290–380 °C and a maximum degradation rate at around 370–380 °C [34,35]. Li et al. [36] reported that PLA in fibrous and nonwoven forms exhibited relatively low thermal stability due to its limited crystallinity and structural characteristics. Despite that, the decomposition proceeds predominantly via depolymerization and random chain scission, leading to the formation of lactide and other volatile products. In contrast, PBS exhibits higher thermal stability, with the onset of degradation usually reported at approximately 320–330 °C and the main decomposition occurring between 300 and 450 °C, with a DTG peak around 380–390 °C. The thermal degradation of PBS proceeds through mechanisms typical for aliphatic polyesters, initiated by random scission of ester bonds, followed by β-elimination (cis-elimination) reactions [37]. These processes result in the formation of unsaturated compounds, oligomers, and volatile degradation products such as carbon dioxide. Among the analyzed polymers, PA1010 exhibits the highest thermal stability, with the onset of degradation typically above 400 °C and the maximum decomposition rate occurring at approximately 480–490 °C [27]. This enhanced thermal resistance is attributed to the presence of strong intermolecular hydrogen bonding and a more stable backbone structure characteristic of polyamides. The thermal degradation of PA1010 involves chain scission accompanied by the formation of volatile products such as amines, hydrocarbons, and nitriles. Unlike aliphatic polyesters, polyamides may exhibit slightly more complex degradation behaviour. Overall, although the thermal degradation mechanisms of PLA, PBS, and PA1010 differ at the molecular level, all three polymers typically exhibit a single main mass-loss step in TGA. The observed increase in thermal stability in the order of PLA < PBS < PA1010 is consistent with the inherent structural characteristics and bonding interactions of these polymers, as widely reported in the literature. Additionally, all fabrics similar to applied polymers exhibited a single-step process in TGA, despite the multi-step chemical mechanism of the neat components. It should be emphasized that the thermal stability of the fabric is influenced not only by the intrinsic properties of the polymer but also by the fibre morphology, which generally results in a lower degradation temperature compared to the corresponding bulk material [38,39]. Minor variations in thermal parameters among samples of the same polymer type suggest a limited influence of compositional differences on the degradation behaviour. However, slightly higher T_5%_ and T_50%_ values observed for selected PBS and PA1010 samples indicate that an increased content of the more thermally stable component or higher structural order (e.g., crystallinity) may enhance thermal resistance. Overall, the results confirm that PA1010-based fabrics exhibit the highest thermal stability, making them suitable for applications requiring elevated processing or operating temperatures, whereas PLA-based fabrics are more prone to thermal degradation.

### 3.2. The Surface and Structure of Fabrics

**Figure 3:**
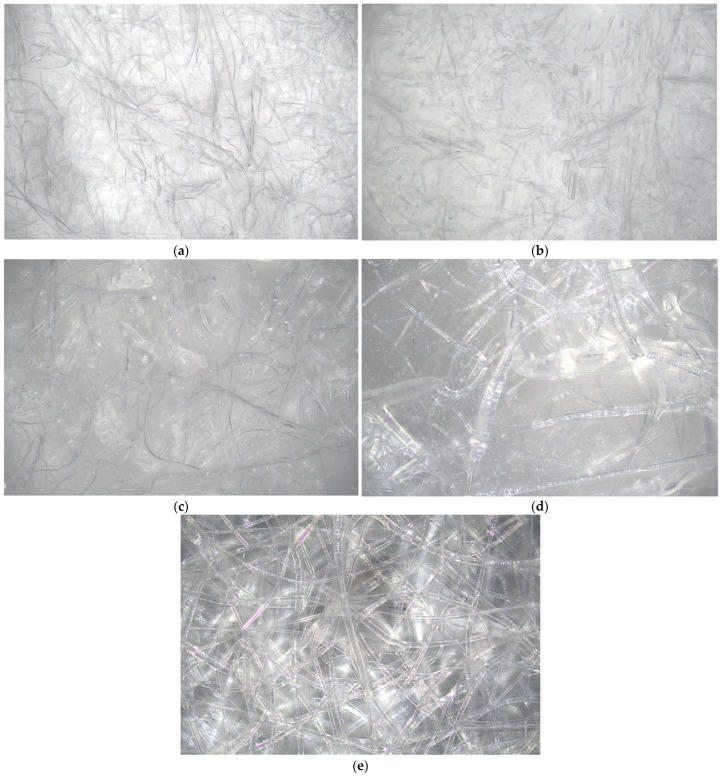

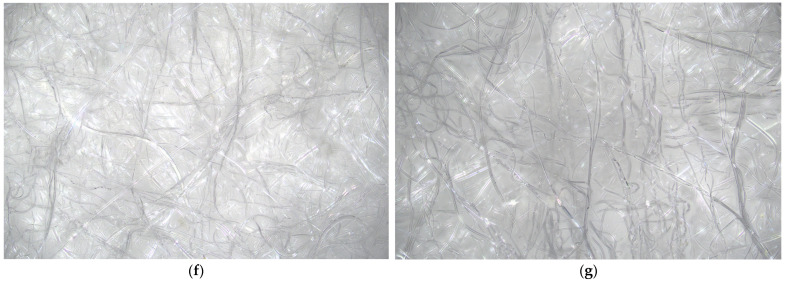
Microscope images for neat fabrics: (**a**) PLA_1, (**b**) PLA_2, (**c**) PBS_1, (**d**) PBS_2, (**e**) PBS_3, (**f**) PA1010_1, (**g**) PA1010_2.

On the other hand, the structure of fabrics was observed using an optical microscope and the results are presented in Figure 3. The magnification of the optical microscopy images was ×200.

The PLA-based fabrics (Figure 3a,b), produced via the melt-blown technique, exhibit a structure composed predominantly of fine microfibres, which are randomly oriented and form a loosely packed, porous network with visible inter-fibre spaces. In the case of PLA_1 (Figure 3a), the structure appeared more heterogeneous, with a broader distribution of fibre diameters and a relatively loose arrangement of fibres. Numerous elongated fibres and locally less densely packed regions could be observed, indicating higher porosity and a less compact structure. The fibres were relatively smooth, with occasional intersections but limited bonding points. In contrast, PLA_2 (Figure 3b) showed a more uniform and compact morphology. The fibres appear slightly more evenly distributed, with a narrower diameter distribution and improved packing density. The structure is more homogeneous, with fewer large voids and a more interconnected network of fibres, which suggests enhanced structural integrity. The observed differences between PLA_1 and PLA_2 may be attributed to variations in PLA content, which influenced fibre formation, diameter distribution, and degree of entanglement. The more compact structure of PLA_2 is expected to contribute to improved mechanical performance and potentially better resin impregnation in composite applications, whereas the more porous structure of PLA_1 may facilitate higher permeability but lower structural cohesion.

The microstructure of PBS-based fabrics is presented in Figure 3c–e. The observed differences in morphology are strongly related to both the polymer content and the fabrication method. The PBS_1 sample (Figure 3c), produced via the melt-blown technique, exhibits a structure composed predominantly of relatively thick microfibres with irregular orientation and a broad diameter distribution. The morphology appears less uniform, with visible fibre bundles and locally compact regions intertwined with more porous areas. The fibres are smooth and elongated, forming a loosely packed network with relatively large voids, which is characteristic of melt-blown materials [40]. In contrast, PBS_2 (Figure 3d) and PBS_3 (Figure 3e), fabricated via electrospinning, display a significantly finer and more homogeneous fibrous structure. The fibres are thinner, more uniformly distributed, and form a highly entangled network. The electrospun morphology is characterized by a higher surface area and reduced pore size compared to the melt-blown sample. In PBS_2 (Figure 3d), the structure appears relatively ordered, with clearly distinguishable individual fibres and moderate packing density. The PBS_3 sample (Figure 3e) exhibits the most developed fibrous network, with a high degree of fibre entanglement and a more compact structure. The increased polymer content likely contributes to improved fibre continuity and more uniform fibre formation, resulting in enhanced structural cohesion. Overall, the transition from melt-blown (PBS_1) to electrospun structures (PBS_2 and PBS_3) leads to a significant refinement of fibre morphology, reduced fibre diameter, and increased structural uniformity. These differences are expected to influence the mechanical properties, surface area, and resin impregnation behaviour of the resulting composites [41,42].

The PA1010-based fabrics (Figure 3f,g) were characterized by a well-developed and relatively compact fibrous structure, with thicker and more continuous fibres forming a cohesive and interconnected network. Compared to PLA and PBS, PA1010 fabrics exhibited lower porosity and higher structural integrity. The difference between PA1010_1 and PA1010.4 lies mainly in the degree of fibre packing and entanglement, with PA1010_2 showing a more compact arrangement. Such morphology may be favourable for effective load transfer and may contribute to improved mechanical performance.

Most importantly, the observed trend in wettability (PA1010 > PLA > PBS) was consistent with the thermogravimetric results, which showed increasing thermal stability in the same order. Fabrics based on PA1010, characterized by the highest decomposition temperatures, also exhibit the most compact and structurally stable fibrous morphology, which promotes both enhanced thermal resistance and hydrophobic behaviour. In contrast, PLA and PBS, with lower thermal stability, form less cohesive fibrous structures, which affects both wettability and mechanical performance.

**Figure 4:**
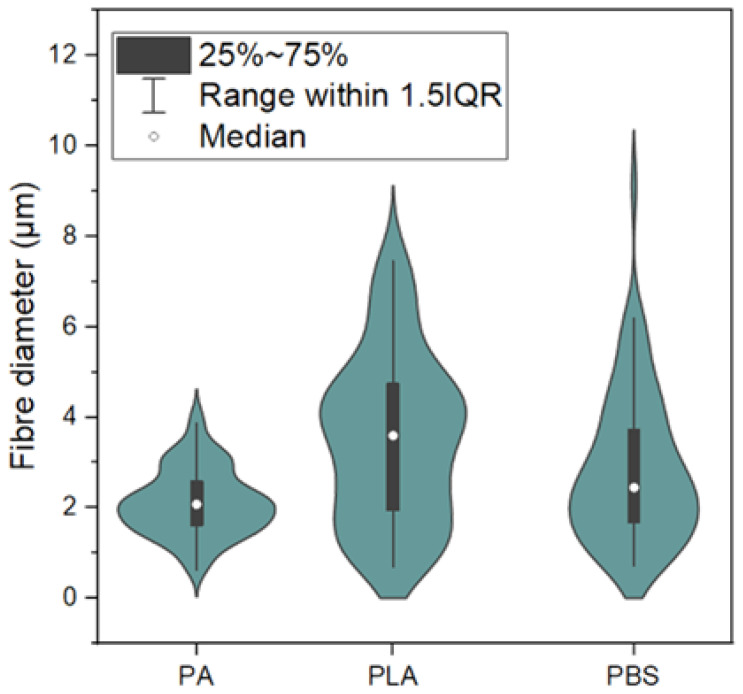
Fibre diameter distribution of PA1010, PLA, and PBS-based nonwoven fabrics presented as violin plots. The black box represents the interquartile range (25–75%), whiskers indicate the range within 1.5× IQR, and the marker denotes the median value.

To further quantify the differences in fibre morphology observed in optical microscopy (Figure 3), the fibre diameter distribution for PA, PLA, and PBS-based fabrics was analyzed and presented in Figure 4.

The results confirmed that PA1010-based fabrics exhibit the narrowest fibre diameter distribution with relatively low median values, reflecting their compact and uniform structure. In contrast, PLA showed a broader distribution with higher variability, indicating a more heterogeneous morphology. PBS-based fabrics exhibited intermediate behaviour, with a wider distribution depending on the processing method, which is consistent with the coexistence of micro- and nanofibrous structures.

These differences in fibre diameter distribution were expected to influence both resin impregnation and mechanical performance of the resulting composites. More uniform fibre diameters, as observed for PA1010, may promote more homogeneous stress distribution and improved interfacial contact with the resin matrix. In contrast, broader distributions, particularly in PLA-based fabrics, may lead to local stress concentrations and less efficient load transfer in the composite. Additionally, the presence of thicker fibres, as observed in PBS_1-type structures, may enhance load-bearing capacity, while finer fibres in electrospun networks increase surface area but may reduce the ability to sustain higher loads. This indicates that fibre diameter distribution plays a key role in balancing strength and deformability in fibrous composite systems. This correlation is further reflected in the mechanical properties of the composites discussed in Section 3.5. The presented fibre diameter distributions provide a quantitative description of the reinforcing structures and were used as a basis for interpreting the mechanical performance of the composites.

### 3.3. The Wettability and Contact Angle (CA) Measurements of Fabrics

The wettability of the fabrics, determined based on contact angle (CA) measurements, was calculated and averaged from 6 to 8 repeated measurements of droplets placed on different areas of the fabric surface. The results were processed and averaged using dedicated software, and the standard deviation, corresponding to the measurement error, was CA ± 2°.

**Figure 5:**
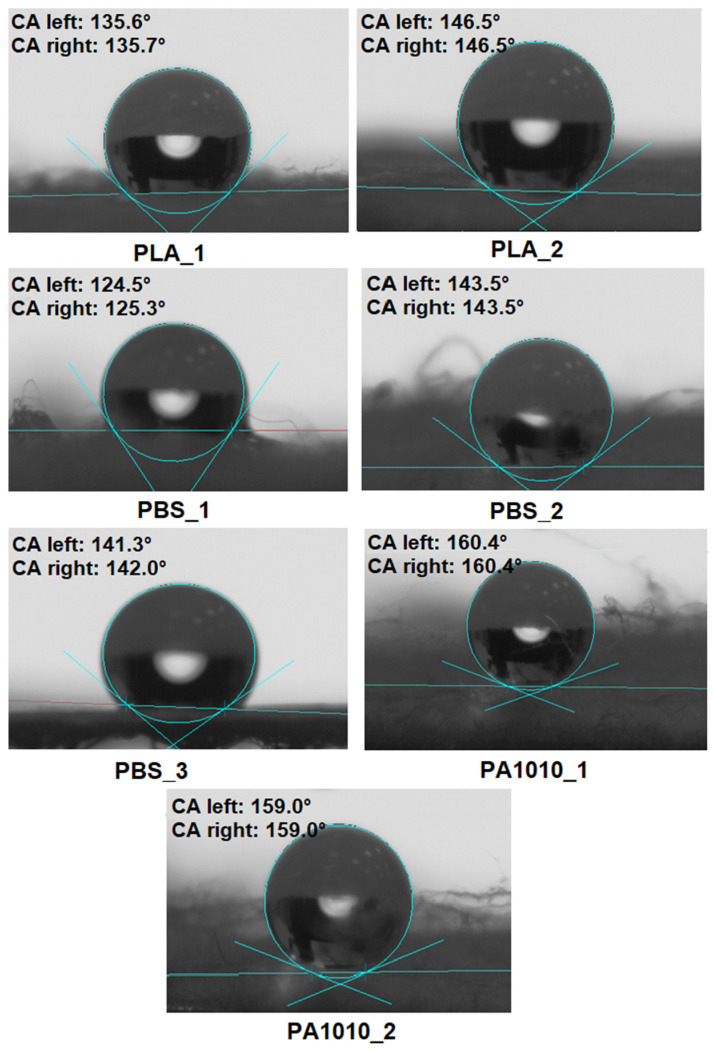
Images of droplets on the surface of fabrics and static water contact angle (WCA).

The wettability of the investigated fabrics is strongly influenced by their surface morphology, fibre diameter, and structural organization, in addition to the intrinsic surface chemistry of the polymer. The observed differences in contact angle can be directly correlated with the microstructure of the fibrous materials. The results of contact angle (CA) measurements are shown in Figure 5.

Analysis of the obtained results indicates that the wettability of examined fabrics was governed by a complex interplay between fibre diameter, porosity, structural uniformity, and fabrication method, with morphology playing a key role in controlling liquid–solid interactions in nonwoven fabrics.

The contact angle (CA) measurements revealed that all investigated fabrics exhibited pronounced hydrophobic behaviour, with WCA values exceeding 120° for all samples (Figure 5).

Among PLA-based fabrics, PLA_1 showed a contact angle of approximately 135.6°, while PLA_2 exhibited an even higher value of 146.5°, suggesting that increased structural uniformity and fibre packing density enhanced hydrophobicity. This effect suggested that improved fibre packing promoted air entrapment within the structure, stabilizing the droplet in a Cassie–Baxter wetting regime. This wetting model describes a state in which the liquid droplet does not fully wet the surface but is partially supported by air pockets trapped within a rough or porous structure, in this case, a fibrous nonwoven material [43]. Despite the inherently moderate hydrophilicity of PLA, the fibrous morphology significantly altered the wetting behaviour. PLA-based fabrics are typically hydrophobic, with inherent water CA ranging from 75° to 85°. Although untreated PLA shows hydrophobic behaviour, structural modifications can produce superhydrophobic surfaces with CA exceeding 150°. On the other hand, treatments such as deep eutectic solvents may reduce the CA, thereby enhancing the material’s wettability [44,45,46].

For PBS-based fabrics, a clear dependence on processing method, structural organization and composition was observed. The melt-blown PBS_1 sample exhibited a contact angle of 124.5°, indicating slight liquid penetration into the porous structure. In contrast, PBS_2 and PBS_3, characterized by more refined and homogeneous fibrous networks (due to the electrospinning process), showed significantly higher CA (~141–143°), suggesting reduced liquid penetration and increased air trapping within the structure. A similar situation to PLA applies to nonwoven materials based on PBS. The WCA of PBS fabrics and films generally indicated moderate hydrophobicity, with CA typically in the range of approximately 78–96° for untreated, smooth PBS surfaces. However, the CA of PBS may vary significantly depending on surface treatment, structure, or blending. For example, melt-blown polypropylene (PP) fabrics, which are highly hydrophobic (CA~140°), become more hydrophilic after blending with PBS, with the CA decreasing to 120–130°, depending on the amount of PBS incorporated [47,48,49].

The PA1010-based fabrics exhibited the highest values of CA (~159–160°), indicating near-superhydrophobic behaviour. This may be attributed to their well-developed, compact fibrous morphology, which effectively promotes air entrapment and minimizes solid–liquid contact area. These values are significantly higher than those observed for both PLA and PBS-based fabrics. Moreover, the PA1010-based fabrics demonstrated a more stable wetting regime compared to PLA and PBS, as evidenced by the highly symmetrical droplet shape and minimal deformation at the contact line. This suggests a stable Cassie–Baxter state, in which the liquid droplet is supported by a fabric solid–air interface within the fibrous structure.

Overall, the results indicate that PA1010-based fabrics exhibit superior hydrophobic performance compared to PLA and PBS, which may contribute to improved resistance to moisture and better interfacial behaviour in composite systems.

This indicates that wettability in fibrous systems should be interpreted as a structural parameter linked to surface topology and air entrapment, rather than as a direct measure of intrinsic polymer polarity.

### 3.4. Monitoring the Curing Process of Bio-Resin Composites

**Figure 6:**
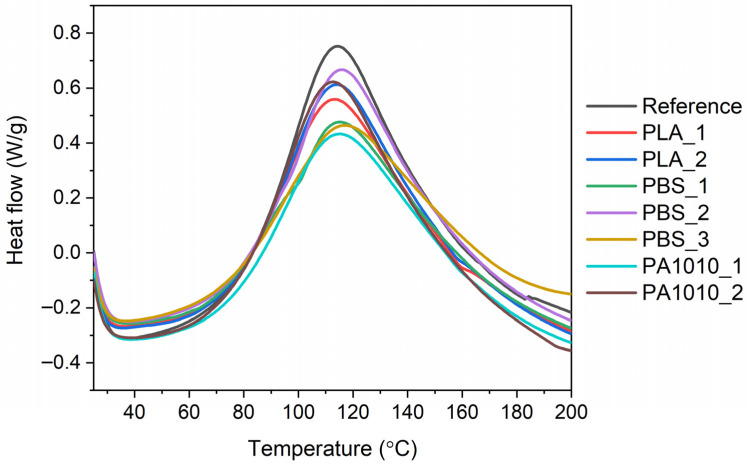
Curing curves of bio-resin composites measured by DSC.

To investigate the curing process of bio-resin composites containing PLA, PBS or PA fabrics, differential scanning calorimetry (DSC) was employed. The summarized results are presented in Table 2 and shown in Figure 6.

The DSC results of the bio-resin composites (Table 2) indicated that the curing behaviour of the laminates from bio-resin and fabrics was influenced by the type of fibrous reinforcement and its chemical nature. The curing curves of the bio-resin laminates reveal a similar overall curing profile for all investigated systems, with a single dominant exothermic peak (Figure 6). The curing process occurred within a similar temperature range (approximately from 38 to 48 °C to 191–200 °C) for all composites, suggesting that the type of fabrics had no significant influence on the reactivity of the bio-resin matrix with the curing agent. The peak curing temperature (T_peak_), understood as the temperature at which the resin curing is the fastest, remained relatively constant (~114–118 °C), indicating that the overall curing mechanism was not significantly altered by the type of reinforcement.

**Figure 7:**
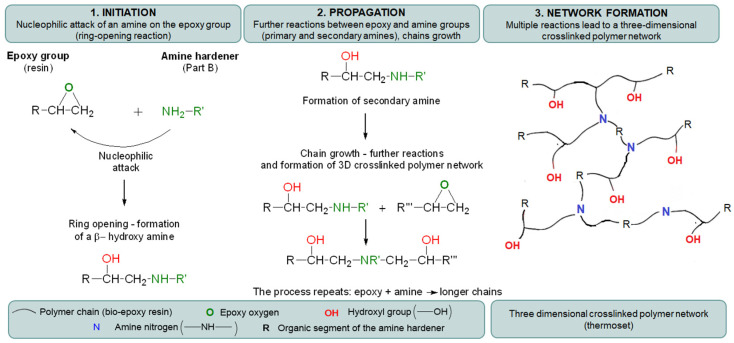
Mechanism of curing of bio-resin composites using amine hardener.

On the other hand, some differences were observed in the enthalpy of curing (ΔH), which reflects the extent of the curing reaction and the amount of heat released during network formation. The bio-based epoxy laminating resin was cured using an amine hardener (Part B), which served as the curing agent. Thus, the curing mechanism proceeded via nucleophilic ring-opening of the epoxy groups by amine functionalities, resulting in the formation of a three-dimensional crosslinked polymer network [50,51] as was shown in the scheme in Figure 7.

The curing of resin involves the initiation of reactions between epoxy and amine groups, followed by chain propagation and the progressive development of a highly crosslinked spatial structure [50,51]. The reference sample, i.e., neat bio-resin, exhibited the highest curing enthalpy (348.8 J/g), while the composites showed varying ΔH values depending on the polymer type in fabrics, which was reflected in the intensity of the peak on the DSC curves of the curing process (Figure 6). PLA-based composites (PLA_1 and PLA_2) showed moderate curing enthalpy values (306.3 and 324.9 J/g), suggesting relatively good compatibility of fabrics with the bio-resin [52,53]. This can be attributed to the presence of polar ester groups in PLA, which may promote interactions with the resin matrix and facilitate curing reactions. The slightly higher ΔH value for PLA_2 may be due to its more compact and homogeneous fibrous structure, which improved resin distribution and interfacial contact. In the case of PBS-based composites, a wider variation in ΔH values was observed. PBS_2 showed a relatively high curing enthalpy (324.0 J/g), while PBS_1 and PBS_3 exhibited significantly lower ΔH (279.3 and 255.3 J/g, respectively). This may result from both structural and chemical factors. PBS, as an aliphatic polyester, has a lower polarity compared to PLA, which may limit interfacial interactions with the resin. Additionally, excessive fibre refinement (PBS_3) or a highly porous structure (PBS_1) may hinder uniform resin distribution, leading to reduced curing efficiency. On the other hand, PA1010-based composites exhibited contrasting behaviour. While PA1010_1 showed moderate curing enthalpy (287.0 J/g), PA1010_2 reached a ΔH value (344.9 J/g) comparable to the reference sample. This can be attributed to the presence of amide groups in PA1010, which enable strong intermolecular interactions, including hydrogen bonding with the resin system. These interactions enhance interfacial adhesion and promote more efficient network formation. Furthermore, the more compact fibrous structure of PA1010_2 likely facilitates better resin impregnation and curing. The presence of larger fibre voids and their uneven distribution may hinder effective resin impregnation and limit the extent of the curing reaction.

Additionally, samples exhibiting the most compact and cohesive fibrous morphology, i.e., bio-resin composite based on PA1010_2, showed one of the highest curing enthalpy values, which is consistent with its superior wettability behaviour (high contact angle). It may also have an effect on the mechanical performance of bio-resin composites. This indicates that the well-organized fibrous network promotes efficient stress transfer and optimal resin–fibre interactions during curing.

Furthermore, the wettability results support this interpretation. Structures that promote a stable Cassie–Baxter regime (e.g., PA1010 and PLA_2) were characterized by a well-defined surface morphology that also facilitated resin distribution during processing. Although high hydrophobicity may intuitively suggest poor wetting, in fibrous systems it often reflects an optimized hierarchical structure that enhances uniform resin infiltration at the microscale [43,54]. From a mechanistic perspective, the obtained results suggest that fibre morphology governs the efficiency of resin infiltration and the development of the fibre–matrix interphase. More compact and structurally integrated fibrous networks promote a more uniform distribution of the resin at the microscale, increasing the effective contact area and facilitating intermolecular interactions during curing. In contrast, highly porous or excessively refined fibrous structures may lead to local resin heterogeneities and reduced crosslinking efficiency. This indicates that the interfacial performance is controlled not only by the chemical nature of the polymer but also by the geometrical and hierarchical organization of the fibrous reinforcement [55].

### 3.5. Mechanical Properties of Bio-Resin Composites

The mechanical performance of the bio-resin composites was further evaluated in terms of tensile properties, hardness, and flexural behaviour, providing a comprehensive understanding of the structure–property relationships governing the analyzed systems. As demonstrated in the previous sections, the morphology of the fibrous reinforcements, including fibre diameter distribution, wettability, and structural cohesion, plays a crucial role in determining resin impregnation and interfacial adhesion. These factors directly affect stress transfer efficiency within the composite and, consequently, its macroscopic mechanical response [56,57,58].

In particular, the fibre diameter distribution (Figure 4) provided quantitative confirmation of the morphological differences observed in optical microscopy. Variations in fibre diameter and distribution width were expected to influence both load-bearing capacity and stress distribution within the composite structure [58,59].

The tensile properties (Table 3) clearly indicated that the mechanical response was strongly dependent on both the intrinsic properties of the reinforcing polymer and the architecture of the fibrous network. The highest tensile strength was observed for PA1010_2 (49.1 MPa) and PBS_1 (42.7 MPa), indicating highly efficient stress transfer between the matrix and reinforcement. In the case of PA1010-based composites, this behaviour can be attributed to the combination of a relatively uniform fibre diameter distribution (Figure 4), compact morphology, and high structural integrity, all of which contribute to reduced stress concentration and improved load distribution [56,57].

The relatively high tensile strength of PBS_1 can be explained by the presence of thicker microfibers, as indicated by the broader fibre diameter distribution. Larger fibre diameters may contribute to increased load-bearing capacity of the reinforcement; however, this effect is inferred from the observed trends and was not directly measured in the present study [59,60]. In contrast, PBS_2 and PBS_3, despite their more homogeneous structure, exhibited lower tensile strength, which may be associated with their finer fibre diameters and highly entangled networks, although the exact contribution of fibre stiffness, fibre–matrix interfacial adhesion, and possible structural defects was not directly quantified [58,60]. This behaviour was consistent with previous studies on fibrous and electrospun composites, where fibre diameter and structural organization were identified as key factors governing stress transfer efficiency and mechanical performance.

In comparison with literature data for bio-resin composites reinforced with natural or synthetic fibres, where tensile strength typically ranges from approximately 20 to 40 MPa, the values obtained in this study (up to 49 MPa) indicate competitive or improved mechanical performance. This suggests that nonwoven fibrous reinforcements derived from biopolymers can serve as an effective alternative to conventional reinforcement systems, particularly when optimized in terms of morphology and structural organization [61].

PLA-based composites exhibited moderate tensile strength (15.3–20.8 MPa), which was consistent with the known brittleness and limited toughness of PLA [53]. The relatively broad fibre diameter distribution observed for PLA suggests a higher degree of structural heterogeneity, which may promote stress localization and reduce overall strength, although this interpretation is based on structural observations rather than direct measurements of local stress distribution [2].

The elongation at break (E_b_) results (Table 3) further highlight differences in deformation mechanisms. The E_b_ was observed for PLA_1 (23.2%), which can be associated with its more heterogeneous structure, allowing for progressive deformation and energy dissipation [3]. In contrast, PBS_3 exhibited very low E_b_ (2.9%), indicating a more brittle response associated with restricted mobility of polymer chains in a dense fibre network.

**Figure 8:**
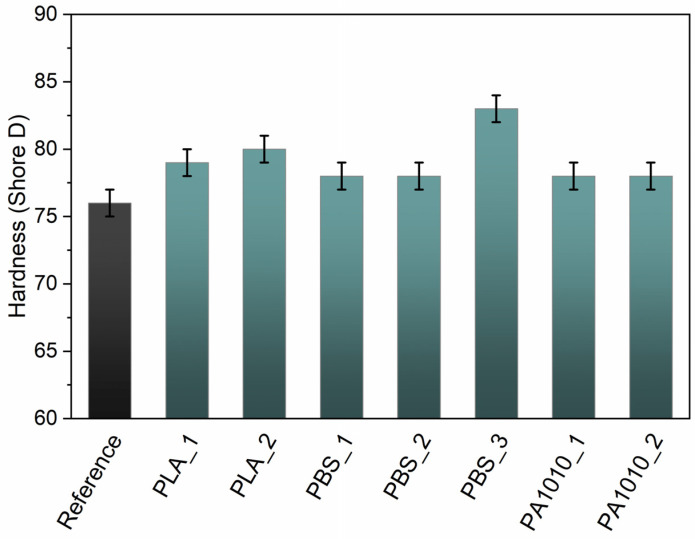
Shore D hardness of bio-resin composites.

The hardness results (Figure 8) are consistent with the trends observed in tensile properties and fibre morphology. PA1010-based composites exhibited the highest hardness, which can be attributed to their compact structure and reduced porosity, limiting localized deformation under indentation [56]. PLA-based composites showed lower hardness, likely due to their higher structural heterogeneity, while PBS-based systems exhibited intermediate behaviour.

The hardness results shown in Figure 8 further emphasized the influence of structural compactness and resin distribution within the composite. Materials characterized by more uniform and densely packed fibre networks exhibited higher resistance to indentation, indicating more effective load transfer at the microscale. In contrast, composites with higher structural heterogeneity and porosity were more prone to localized deformation, which resulted in lower hardness values. This behaviour suggested that hardness was not only governed by the intrinsic properties of the polymer but also strongly depended on the quality of fibre–matrix interaction and the degree of impregnation of the fibrous structure.

The flexural properties (Table 4) further confirmed the observed structure–property relationships. PA1010-based composites exhibited the highest stiffness and flexural strength, which can be attributed to their compact morphology and efficient stress transfer under bending conditions [56,57].

PBS-based composites displayed more variable flexural behaviour, reflecting differences in fibre diameter and processing method. Systems with larger fibre diameters showed improved load-bearing capacity, while those with finer fibres exhibited reduced stiffness despite higher structural uniformity [59,60].

PLA-based composites exhibited lower flexural properties, consistent with their intrinsic brittleness [53]. However, improved fibre organization in PLA_2 resulted in enhanced performance, confirming the importance of structural uniformity and interfacial interactions [57].

The flexural strain at break (εfM) provides additional insight into the deformation behaviour of the composites under bending. Materials with more heterogeneous and less compact structures, such as PLA-based systems, tend to exhibit higher strain values, indicating increased flexibility. In contrast, more compact and structurally integrated systems, particularly PA1010-based composites, showed lower strain, corresponding to higher stiffness and reduced deformability.

This trend was consistent with tensile behaviour, where increased structural heterogeneity resulted in higher elongation at break, confirming the strong relationship between fibre morphology and deformation mechanisms.

Overall, the mechanical behaviour of the analyzed resin composites was governed by a synergistic interplay between fibre diameter distribution, structural morphology, and intrinsic polymer properties, which was consistent with recent studies on electrospun and fibrous polymer systems [56,57,58,59]. These results highlighted a fundamental trade-off between structural uniformity and load-bearing capacity, where finer fibres improve homogeneity but may reduce mechanical strength, while thicker fibres enhance strength at the expense of structural uniformity. From an application perspective, these findings suggested that the selection of fibre architecture should be tailored depending on whether stiffness, strength, or deformability is the primary design requirement.

The results further allow the formulation of a semi-quantitative structure–property relationship. Fibre systems characterized by narrower diameter distribution and higher packing density (e.g., PA1010_2) exhibited improved stress transfer efficiency and higher tensile strength, whereas systems with broader distributions or highly heterogeneous structures showed increased deformation capability but reduced strength. Additionally, the presence of thicker fibres contributes to load-bearing capacity, while finer fibres increase surface area but may limit effective stress transfer due to reduced individual fibre stiffness. This trade-off between structural uniformity and load-bearing capacity represents a key design parameter in fibrous composite systems.

It should be noted that the proposed relationships between fibre morphology and mechanical performance are based on observed correlations across the investigated systems. Parameters such as fibre intrinsic mechanical properties, fibre–matrix interfacial strength, and void content were not directly measured in this study. Therefore, the presented interpretation should be considered as a semi-quantitative description of structure–property relationships rather than a definitive mechanistic model.

### 3.6. Thermal Stability of Bio-Resin Composites

**Figure 9:**
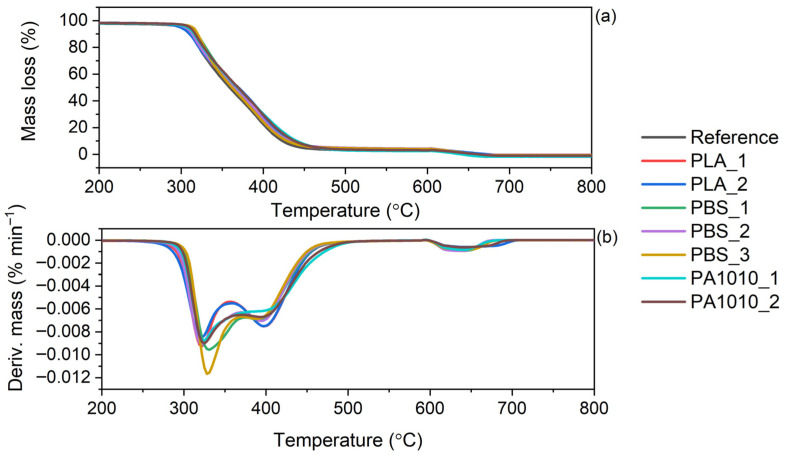
TG and DTG curves of bio-resin composites: (**a**) TG curves, (**b**) DTG curves.

The thermal stability of bio-resin composites containing fibres based on polymers such as PLA, PBS, and PA1010 was investigated and evaluated based on the temperatures at which specific mass losses occurred, namely 5% (T_5%_), 10% (T_10%_), and 50% (T_50%_). The obtained results are summarized in Table 5. The thermogravimetric (TG) and derivative thermogravimetric (DTG) curves are shown in Figure 9.

The TGA of the bio-resin composites (Figure 9) revealed that all laminates exhibited a similar degradation profile. This indicated that the thermal decomposition process was primarily governed by the resin matrix, with the reinforcing fabrics influencing the degradation behaviour to a lesser extent. The onset of degradation (T_5%_) for all composites occurred in a relatively narrow range of 299–313 °C, which was comparable to the reference neat resin (302 °C). This suggested that the incorporation of different fibrous reinforcements did not significantly alter the initial thermal stability of the system. However, slight variations may be observed depending on the type of polymer reinforcement.

Among the investigated systems, PBS-based laminates exhibited slightly higher T_5%_ (up to 313 °C for PBS_3), indicating slightly improved initial thermal stability compared to PLA-based composites (Table 5). This trend is consistent with the TGA results of the neat fabrics, where PBS showed higher thermal stability than PLA. In contrast, PA1010-based composites did not show a substantial increase in T_5%_, despite the high thermal stability of the neat PA1010 fibres (T_5%_ > 400 °C). This suggested that the early-stage degradation of the laminates was dominated by the resin matrix rather than the reinforcement.

More pronounced differences were observed at higher temperatures. The temperature corresponding to 50% mass loss (T_50%_) and the temperature of maximum degradation rate (T_DTG_) showed a clear influence of the reinforcing fibres. Laminates containing PA1010 exhibited the highest T_DTG_ of approximately 481–483 °C, closely matching those of the neat PA1010 fibres. Similarly, PBS-based laminates showed intermediate T_DTG_ (~416–418 °C), while PLA-based composites achieved the lowest T_DTG_ (~379–380 °C) due to the lowest thermal stability of PLA fabrics.

The obtained results confirmed that improved fibre morphology enhanced both wettability control and thermal performance of the resin composites. The highly compact fibrous structure and high hydrophobicity (~160°) of PA1010 fibres, which promote uniform resin distribution and strong interfacial integrity, resulted in more controlled thermal degradation of laminates and reflected the intrinsic thermal stability of the reinforcing polymer.

## 4. Conclusions

This study demonstrates that fibre morphology and structural organization are the key parameters governing the performance of bio-based composite laminates reinforced with biopolymer nonwoven fabrics. Among the investigated systems, PA1010-based reinforcements, characterized by a compact and uniform fibrous architecture, provided the most efficient stress transfer, leading to improved mechanical performance, including the highest tensile strength, flexural properties, and hardness. In contrast, PLA-based composites exhibited lower strength but enhanced deformability, while PBS-based systems showed morphology-dependent intermediate behaviour.

The curing process, analyzed by DSC, was primarily controlled by the resin matrix; however, fibre type and morphology influenced curing efficiency, indicating the importance of interfacial interactions and resin distribution within the fibrous network.

Thermogravimetric analysis confirmed that the thermal stability of all laminates was governed mainly by the bio-resin matrix, whereas the degradation behaviour at higher temperatures was strongly dependent on the reinforcing polymer. PA1010-based laminates exhibited enhanced thermal resistance and a more controlled degradation process associated with improved fibre–matrix integration.

Based on the obtained results, a morphology-controlled performance model can be proposed, in which:

- fibre diameter distribution governs stress transfer efficiency,
- structural compactness controls resin impregnation and interfacial contact, and
- wettability reflects the hierarchical organization of the fibrous structure rather than solely the intrinsic surface chemistry.

This model provides a practical guideline for tailoring fibre architecture in order to optimize the performance of bio-based composite laminates.

Overall, the results establish a clear structure–property relationship, demonstrating that optimizing fibre diameter distribution, morphology, and interfacial compatibility is essential for designing high-performance, sustainable composites. This work highlights the strong potential of recycled biopolymer fibrous materials as advanced reinforcement in next-generation bio-based laminates.

## Figures and Tables

**Table 1:** Thermal properties of fabrics determined by thermogravimetric analysis (TGA).

Fabrics	T_5%_ (°C)	T_10%_ (°C)	T_50%_ (°C)	T_DTG_ (°C)
PLA_1	298	322	367	379
PLA_2	305	328	368	380
PBS_1	348	365	403	416
PBS_2	353	368	405	417
PBS_3	355	369	406	418
PA1010_1	406	430	465	481
PA1010_2	420	438	472	483

T_5%,_ T_10%_, T_50%_—temperature at which 5%, 10% and 50% mass loss of the sample was observed, T_DTG_—temperature of the peak of the main decomposition process.

**Table 2:** Differential Scanning Calorimetry (DSC) results of bio-resin composites (SD: T ± 2 °C; ΔH ± 1.2 J/g).

Composites	Curing Temperature (°C)	T_peak_ (°C)	−ΔH (J/g)
Reference	42–200	116	348.8
PLA_1	40–200	115	306.3
PLA_2	38–200	116	324.9
PBS_1	46–200	117	279.3
PBS_2	42–198	118	324.0
PBS_3	41–191	118	255.3
PA1010_1	48–200	116	287.0
PA1010_2	42–198	114	344.9

**Table 3:** Tensile strength (TS) and elongation at break (E_b_) of bio-resin composites.

Composites	TS (MPa)	E_b_ (%)
Reference	16.2 ± 0.9	14.0 ± 2.1
PLA_1	20.8 ± 1.1	23.2 ± 3.1
PLA_2	15.3 ± 0.8	11.4 ± 1.0
PBS_1	42.7 ± 2.2	11.1 ± 1.1
PBS_2	15.9 ± 0.8	6.5 ± 0.3
PBS_3	33.7 ± 1.6	2.9 ± 0.2
PA1010_1	25.2 ± 1.2	11.9 ± 1.4
PA1010_2	49.1 ± 2.3	9.9 ± 1.0

**Table 4:** Flexural properties of bio-resin composites: flexural modulus (Ef), flexural strength (δfM), and flexural strain at break (εfM). Values are presented as mean ± standard deviation.

Composites	Ef (MPa)	δfM (MPa)	εfM (%)
Reference	520 ± 49	17.8 ± 2.2	53.7 ± 4.7
PLA_1	1630 ± 60	23.9 ± 3.1	1.3 ± 0.2
PLA_2	1670 ± 65	39.0 ± 4.2	1.6 ± 0.2
PBS_1	1470 ± 53	37.0 ± 3.9	3.6 ± 0.3
PBS_2	903 ± 78	37.9 ± 3.7	5.0 ± 0.3
PBS_3	1310 ± 55	60.6 ± 7.7	5.0 ± 0.4
PA1010_1	1870 ± 64	77.4 ± 7.5	3.8 ± 0.2
PA1010_2	870 ± 99	52.6 ± 4.8	4.8 ± 0.3

**Table 5:** Thermal properties of bio-resin composites determined by TGA.

Fabrics	T_5%_ (°C)	T_10%_ (°C)	T_50%_ (°C)	T_DTG_ (°C)
Reference	302	311	358	328
PLA_1	302	312	368	379
PLA_2	299	310	370	380
PBS_1	311	319	363	418
PBS_2	304	313	364	417
PBS_3	313	320	361	416
PA1010_1	307	315	367	481
PA1010_2	310	316	368	483

T_5%,_ T_10%_, T_50%_—temperature at which 5%, 10% and 50% mass loss of the sample was observed, T_DTG_—temperature of the peak of the main decomposition process.

## Data Availability

The original contributions presented in this study are included in the article. Further inquiries can be directed to the corresponding authors.
